# From Work Ability Research to Implementation

**DOI:** 10.3390/ijerph16162882

**Published:** 2019-08-12

**Authors:** Juhani Ilmarinen

**Affiliations:** Juhani Ilmarinen Consulting Ltd., Ruuvitie2, 01650 Vantaa, Finland; juhani.ilmarinen@jic.fi

**Keywords:** work ability index (WAI), work ability concept, intervention research, knowing–doing gap, implementation

## Abstract

Work ability research started in Finland in the 1990s due to the challenges of work force aging. The employment rates of older workers (55+) were below 40% and early retirement and work disability rates were rather common in many European countries. The work ability concept and methods were developed and broad international research activities started in the 1990s. A comprehensive promotion model for work ability was created aiming to prevent work ability from declining during aging. However, to be able to impact the work ability is a complicated and difficult task, and requires effects on human resources, work arrangements, and management. Therefore, only a limited number of intervention studies have shown an improvement of work ability during aging. This article introduces some possibilities regarding how to make work ability interventions more successful.

## 1. Background

Population and work force aging were the main reasons for starting the work ability research in the early 1980s, and a comprehensive concept for occupational health research was developed by the Finnish Institute of Occupational Health (FIOH) [1]. The employment rates of older workers (55–64 years) in many European countries were close to 41% in 2003, early retirement options were widely used, and only a minority of older workers retired at mandatory retirement ages. Although the situation has improved, and many countries have carried out pension reforms, severe concerns remain regarding how the older workers can or will work longer. The current changes in working life, globalization, digitalization, and new technology, as well as the requirements for better quality and productivity, increase the challenges for everybody, but especially for older workers and employees worldwide. Excellent state-of-the-art books are available [2,3]. Additionally, we are facing new challenges of a multi-age workforce nowadays [4]. Therefore, the human ability to work during the life course and aging remains in the focus of employment and social policy. Longer and better working lives will be a continuous challenge for our societies [5]. 

**Figure 1:**
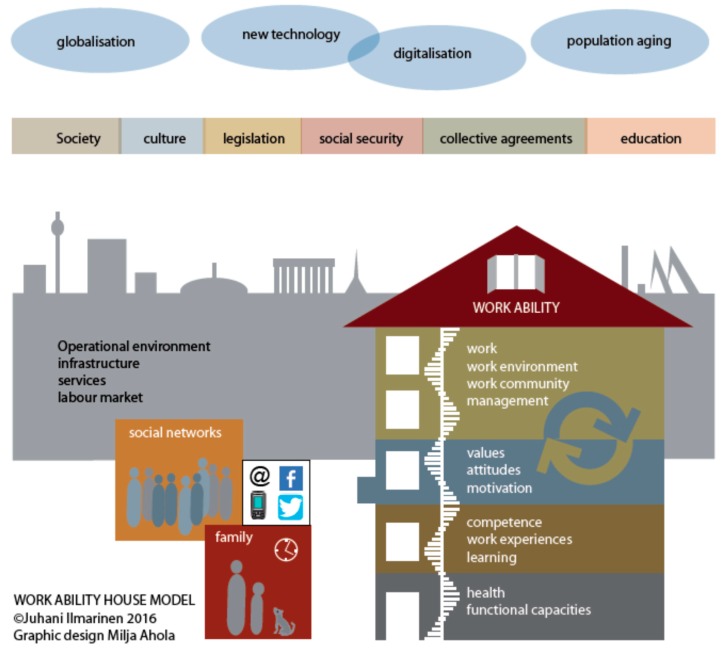
The work ability house model. The floors of the house, as well as family and social networks, indicate dimensions that affect work ability. Management and leadership skills on floor 4 have the strongest effect on work ability. In the third floor, the single factors like appreciation, trust, fair treatment, and support effect workplace well-being. Sustainable balance between factors of work and human resources creates good work ability.

The basis for the work ability research and construction of the work ability index (WAI, which can be found from the Aging Worker Supplement of SJWEH [6], and the validation of the WAI in the 11-year follow-up study [7]). An updated user manual for WAI from 2012 is available from the bookstore of the FIOH. The model to promote the sustainable work ability and work well-being during aging is based on the work ability–house model (Figure 1), which describes the requirements for a person–environment (PE) fit. 

Because successful interventions to promote work ability are a demanding process, I have focused my paper, based on my experiences, to give researchers and practitioners some ideas on how to improve the effectiveness of workplace interventions. A good basis for work ability interventions is available from Oakman et al. [8].

A history of work ability has been introduced earlier [9], but here is a short summary of the main activities during the last 30 years:

Between 1980 and 1989, the evolution of work ability as a new paradigm compared to work disability was started by FIOH. It included the development of the work ability index (WAI), as well as a follow-up study of Finnish municipal employees (1981–2009) [7].

Between 1990 and 1999, the promotion concept of work ability was developed based on the results of an 11-year follow-up study [7]. WAI in occupational health services was implemented. The internationalization of the work ability concept and WAI was started (The Netherlands, Austria, Germany). In all, 17 international work ability conferences, symposia, and workshops were organized by the International Commission of Occupational Health (ICOH) and the International Ergonomic Association (IEA) between 1990 and 2018. Several books and proceedings of international research activities have been published since 2002. The WAI was translated into over 30 languages.

Between 2000 and 2009, the concept called “work ability house” was created based on the Finnish National Survey of work ability [10]. The implementation of research findings into practice were forced. Work ability training, coaching, and counselling were started in Germany [11] and Austria [12]. In work ability coaching, about 1300 persons have been trained, and from them, more than 500 persons are active service providers of work ability A WAI network was established in Germany. In the Netherlands, wide national activities were carried out by Blik op Werk to improve the publicity of work ability. Research activities were also started in Business, Work and Ageing, Swinburne University of Technology, Melbourne, Australia.

In 2010, the work ability house model was updated (Figure 1). New instruments were published, such as Work Ability Plus in Austria [11], and Work Ability 2.0 in Finland [13]. A work ability graduate course was started in the medical faculty of the University of Vienna, Austria. An institute of Work Ability was established in Germany. A comprehensive catalog of seven work ability instruments were published in Germany by Initiative Neue Qualität der Arbeit (https://www.inqa.de/EN/Home/home.html).

Several scientific papers were published from the Finnish Longitudinal Study of Ageing Municipal Employees (FLAME) study in collaboration between FIOH, University of Jyväskylä, and University of Tampere, Finland. The collaboration between occupational health research and gerontology had started.

## 2. Research Activities on Work Ability

Most of the research activities of work ability has been focused in occupational health research, epidemiology, and ergonomics, and recently, in occupational gerontology. Our understanding of factors affecting work ability has been improved significantly. The interactions between human resources and work are intensive and dynamic. These interactions are changing due to the life course and aging. The balance between the human resources (health and functional capacities, competence, values, attitudes, and motivation) and work (demands, work arrangement, and management) is crucial. A poor balance decreases the work ability in physical, mental, and mixed work, both among men and women [7]. This is probably the main reason why the work ability seems to decline worldwide during aging. An important research question remains unanswered: Is the main reason for poor balance predominantly due to problems in work organization and in management, or the decline on human resources due to aging? Most of the studies show that both reasons are responsible. Additionally, the family and close community also affect the balance between human resources and work. Therefore, the promotion of work ability becomes even more comprehensive and complex. The promotion of work ability is a new area of potential development for work life developers.

The complexity of interactions explains why many intervention studies for the promotion of work ability have been less promising than expected. The recent meta-analysis of 17 randomized control trials showed a small positive effect, suggesting that workplace interventions might improve work ability [8]. The authors recommend high quality studies to establish the role of interventions on work ability. I do agree that better studies are needed, although the situation in dynamic and changing work organizations makes the realization of proper interventions more difficult than before. In the following, I will introduce some reasons, based on my experiences, that could be taken into consideration to make interventions more effective.

## 3. Knowing–Doing Gap

**Figure 2:**
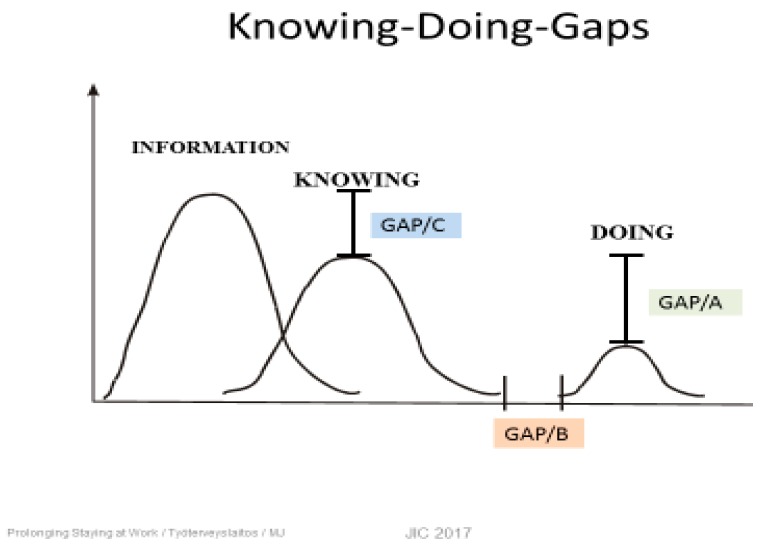
The knowing–doing gap model [14].

Behind the challenge of effective interventions is the knowing–doing (K-D) gap (Figure 2). The K-D gap indicates that the knowledge about the problems in workplaces is extensive compared to how we are able to turn knowledge into action [14]. Every workplace survey increases our knowledge of factors that (Gap C) should be improved to promote the work ability. It seems to be much easier to improve our knowledge than to carry out successful actions (Gap A). Additionally, the time gap gets longer before proper actions happen (Gap B). Therefore, the workers and employees will be frustrated recognizing that, again, nothing has been changed or improved. We should pay much more attention to doing and increase our competences for implementation processes of scientific knowledge at workplaces.

According to my experiences of intervention studies over decades in several countries, at least three main reasons explain why the K-D gaps are growing. The first one is the lack of prioritization of the actions needed. For example, a work ability survey will easily produce a long list of factors that have negative relationships to work ability. Changing all the significant variables is not possible or feasible. Therefore, prioritization is needed. The next question is: Who is going to decide about the prioritization of measures? My opinion is that the steering group making prioritization should be representatives of the organization (management, HR management, foremen, workers and employees, occupational health and safety officer, other preventive staff members). The next question is: How should they prioritize? It should be based on dialog, where everybody in the steering group can give and explain his or her own arguments. An external facilitator takes care that no one can dominate; everyone’s comments will be noticed according to the rules of dialogue; and finally, a consensus will be created. This procedure is not easy and demands a new culture of communication within the steering group and company. In best cases, a long list of necessary measures can be reduced markedly, and the implementation becomes more feasible.

The second reason for less-effective interventions could be the low participation rates of the people involved. Often the targets are to improve human resources through behavioral changes. For example, improving physical fitness using exercise might interest mostly those who are already active compared to those with more passive habits. The effects of exercise should be significant before effects on work ability can be expected. If only 60% of the intervention group improve their fitness, the 40% who are more passive dilutes the effects of the intervention group markedly. The same happens in competence training. Participation rates in learning new skills and competencies is seldom 100%. The same is true for the training of supervisors. There is often a lack of evidence that the training has been effective. The most difficult task is to change the attitude and behavior of supervisors and foremen. Therefore, at least regarding what should be controlled, is how actively the intervention group has participated in the training. If we accept only those who have been affected by the training in the intervention group, the improvements of their WAI can be significant compared to a control group [15].

The third concern is the outcome variable, which should be sensitive enough for changes. The WAI has been widely used as an outcome for interventions. Originally, the WAI was constructed so that health-related items played an important role in scoring the individual WAI. In other words, if the intervention has a significant effect, the WAI will probably improve. However, without significant health effects items 3, 4, 5, and 6, the potential for improvement is rather limited. On the other hand, improvements in management skills and work arrangements should be powerful enough to improve WAI, but it is not easy to improve managerial skills so significantly that the knowledge is transferred into practice. WAI as an outcome variable requires significant improvements in both the health behavior of employees and the leadership behavior of supervisors. In summary, WAI is a very challenging outcome to achieve for interventions, especially among older workers who easily face the age-related changes in personal resources and health. Besides the WAI, broader measurements of outcomes are often necessary [7].

## 4. Work Ability 2.0

For the large Good Work–Longer Career Program of the Finnish Technology industry (2010–2015), new methods to evaluate work ability were developed [16]. The survey method (Work Ability Personal Radar) focused on the dimensions of the work ability house model (Figure 1). Altogether, questions covered four dimensions within the house and two outside, namely family and close community. Additionally, four items of the original WAI were also included (see Ilmarinen et al. [7]). The items were chosen such that each of them could be used as an outcome variable of concrete action. For example, in the dimension of work, question 13 is the following: Do you get feedback from your supervisor about your work performance (scale 0–10)? When the intervention is focused on improving the feedback culture of supervisors, the outcome will directly indicate how successful the measures were.

The second instrument of Work Ability 2.0, namely the Work Ability–Company Radar, is directly focused toward making the interventions more successful. With the help of this method, the actions will be prioritized and a concrete plan will be made. Both prioritization and an implementation plan are created with the help of a dialog process among a representative steering group. Only 1–3 targets with the highest priorities will be taken for interventions, and the intervention should focus on only one dimension at time (like health or work). This process follows the guidelines of the Metal Age project [17]. The combination of survey and prioritization makes the interventions feasible and effective. Our experiences from Finland (technology industry, about 100 companies) and from Germany (manufacturing industry, traffic, service and hospitals) are promising. The challenge is to create a company culture that is positive for the dialogue and decision-making process. An external, independent facilitator is often needed in the beginning to support the process. The motto of the Work Ability 2.0 is: doing less but the most important improvements. 

## 5. Future Challenges of Work Ability

The comprehensive, dynamic concept of work ability offers possibilities for work organizations to support longer and better working lives. Work ability management is a new potential area of development for supervisors, covering both health and age management. As soon as work ability management becomes one of the core functions of supervisors, the implementation of survey results will be more effective. The commitment of supervisors toward work ability management can be improved using annual evaluation of their results. In Finland, about 30% of supervisors are responsible for work ability management [18]. The challenge is to give them enough time, resources, and personnel for implementations.

Work ability should also be on the agenda of social partners. Collective agreements are welcome because both employers and employees are the winner; better work ability and workplace well-being leads to better productivity, which is a win-win situation. The Finnish Program in the Technology Industry was based on an agreement between the Employer Association and the four largest trade unions; in Germany, the work ability project by a private bus company in the city of Hamburg was based on a similar agreement [19]. Work ability could also be a cornerstone for national policy. In Finland, the work ability was anchored in the Occupational Health (2002) and Safety Acts (2003). The Finnish National Programme of Ageing Workers (1996–2002) and the following pension reform improved the employment rate of older workers and attitudes towards aging. In Germany, a large-scale national program INQA (The Initiative New Quality of Work) has been carried out since 2010. In Austria, several large programs are supported by ministries and social insurance organizations. Work ability methods have been widely used in these programs. 

Today’s trend in several European countries is the improvement of workplace well-being. Workplace well-being can be conceptualized in many ways, but my impression is that it should emphasize the qualitative aspects of work ability. For example, if the balance between work and human resources creates positive effects on values, attitudes, and motivation of the staff, both the work ability and workplace well-being will be improved. Indicators for a better workplace well-being can be found in the updated work ability house model (third floor), which utilizes appreciation, trust, fair treatment, and support. In my understanding, workplace well- being cannot be created without work ability (see the legend for Figure 1).

The discussions in the scientific committee Ageing and Work (ICOH) in the beginning of 2000 strongly supported the need to bridge the gap between occupational health research and gerontology [20,21]. One important future aspect of work ability research would be occupational gerontology. Our 28-year follow-up study indicated that work ability before retirement had long-term effects on the activities of daily living [22]. If the WAI was excellent or good before retirement, a major proportion of the older senior citizens later at ages 73–85 years were able to enjoy disability-free, independent living. Successful promotion of WAI has long-term effects and can indirectly affect the aging process.

Therefore, there are common motivations toward understanding the role of work life and the transfer to the third age. In occupational gerontology, the scientist could develop a method that would take into account both work-related aspects and aspects of daily living, such as that suggested by Nygård and Rantanen [23]. Investments for a disability-free third age should be done during the working life.
